# 767 Medical Decision Making in the Burn Unit: A Qualitative Study

**DOI:** 10.1093/jbcr/irac012.320

**Published:** 2022-03-23

**Authors:** Monica L Gerrek, Marcie A Lambrix, Anna D Goff, Charles J Yowler

**Affiliations:** MetroHealth System; CWRU, Cleveland, Ohio; Case Western Reserve University, Cleveland, Ohio; Case Western Reserve University, MetroHealth System, Cleveland, Ohio; MetroHealth/Case Western Reserve University, Cleveland, Ohio

## Abstract

**Introduction:**

In 1973, Dax Cowart was severely burned as a result of a car explosion. He spent the next 46 years advocating for patients’ rights to decline treatment as he felt his repeated wishes to do so and be allowed to die were ignored by his burn providers. Due in large part to his advocacy, many strongly believe that burn providers are problematically paternalistic and force unwanted treatment upon capacitated, autonomous patients (e.g., Hurst et al 2014). Unfortunately, there are only a few attempts to provide the perspectives of other severely burned patients (e.g., Brewster et al 2006 and Gerrek 2018). The purpose of this qualitative study was in part to gain further understanding of the views of severely burned patients regarding medical decision making during the acute phase of their injury.

**Methods:**

Building on Brewster et al. and Gerrek, our team created a semi-structured interview questionnaire. We followed standard IRB research study protocols and then identified adult individuals with 2^nd^ and 3^rd^ degree thermal injury minimum 30% TBSA burns for recruitment. Though 20 patients met the criteria, we completed 5 (26%) interviews (3 females, 2 males).

**Results:**

All interviewees discussed ways in which they struggled to make informed autonomous decisions for weeks or even months post-injury and felt dependent on the burn team to lead them through treatment decisions.

*“[I]t's all new to me … obviously I've never been burnt like this before. I will assume it's similar for most of the people … when you wake up and find out how badly you were burned, and maybe you try to move and you figure out how bad you're hurting, you don't really make the decisions for yourself. I kinda put myself in their hands … I figured that's my best shot, to listen to them and let them do what they need to do. Really, I didn't want to make any decisions. I just listened to them and put my trust in them that they were doing the best they could for me”. (P2*)

None of the interviewees, not even those who underwent amputation, felt the team acted in an inappropriately paternalistic manner. In fact, as a whole, they believed the team only did procedures that were absolutely necessary for their survival and wellbeing.

*“I believe that they made all the right decisions surgery wise and everything.” (P5*)

**Conclusions:**

This study supports the limited but important work of the perspectives of severely burned patients’ regarding, among other things, how medical decisions were made during the acute phases of their injury. It shows that patients trusted their providers to make and encourage life-saving, wellbeing enhancing decisions during the early stages of treatment when patients are not sure they are capacitated and autonomous. Furthermore, the positive feelings patients had about those decisions did not change over time.

